# Parental Perspectives on a Trial Using Waived Informed Consent at Birth

**DOI:** 10.21203/rs.3.rs-3487820/v1

**Published:** 2023-10-30

**Authors:** Anup Katheria, Georg Schmolzer, Brenda Law, Bradley Yoder, Erin Clark, Walid El-Naggar, Ana Morales, Rebecca Dorner, Benjamin Mooso, Wade Rich, Farha Vora, Neiil Finer

**Affiliations:** Sharp Mary Birch Hospital for Women & Newborns; Royal Alexandra Hospital; Royal Alexandra Hospital; University of Utah; University of Utah School of Medicine; Sharp Mary Birch Hospital for Women & Newborns; UC San Diego; Sharp Mary Birch Hospital for Women & Newborns; Loma Linda University Children’s Hospital; Sharp Mary Birch Hospital for Women & Newborns

## Abstract

**Objectives:**

To determine parental perspectives in a trial with waived consent.

**Study Design:**

Biological parents of non-vigorous term infants randomized using a waiver of consent for a delivery room intervention completed an anonymous survey after discharge.

**Results:**

121 survey responses were collected. Most responding parents reported that this form of consent was acceptable (92%) and that they would feel comfortable having another child participate in a similar study (96%). The majority (> 90%) also reported that the information provided after randomization was clear to understand future data collection procedures. Four percent had a negative opinion on the study’s effect on their child’s health.

**Conclusions:**

The majority of responding parents reported both acceptability of this study design in the neonatal period and that the study had a positive effect on their child’s health. Future work should investigate additional ways to involve parents and elicit feedback on varied methods of pediatric consent.

## Introduction

Obtaining consent for interventional trials in newborns is difficult as many therapies for newborns target only a subset of the population (e.g., preterm or term newborns who are non-vigorous at birth) and eligibility is unpredictable prior to birth.^1^ Conversely, some eligible pregnancies may move towards immediate or urgent delivery making the consent process difficult or inappropriate as well as impracticable. These pregnancies are frequently at higher risk for morbidities or death due to lack of timing for important prenatal therapies such as antenatal steroids or may occur in women that have had less access to prenatal care due to socio-economic status. ^2^

It is critical for researchers to include all eligible populations to ensure that study interventions have a high degree of external validity and generalizability. Antenatal consent has been shown to limit the enrollment of the sickest infants by limiting the opportunity to obtain consent from mothers with inadequate prenatal care and/or emergent deliveries. ^2^ This results in reduced generalizability by excluding some of the sickest infants which represent a population that could potentially benefit the most from the data gathered in such studies.^3^

One approach is to ask Institutional Review Boards (IRBs) for waivers of antenatal consent in situations in which such antenatal consent is challenging. In a prior randomized trial conducted by our group, some centers were approved to approach families after a delivery room intervention for consent for ongoing data collection. ^4^ The centers who approached families after delivery were noted to have a higher consent rate for this future data collection.^5^ However, the percentage of eligible women/newborns enrolled using waiver at these sites varied from 25 to 90 percent of eligible subjects, suggesting that even with the waiver, centers did not randomize all eligible infants.

Another type of waivered trial design that could include all eligible subjects is a cluster randomized crossover design, whereby hospitals are randomly assigned to one intervention for a specified period then crossover to the other intervention for a second specified period. ^6^ We applied this trial design to determine if umbilical cord milking (UCM) for non-vigorous infants could improve outcomes when compared to immediately clamping and cutting the umbilical cord at birth (MINVI trial).^7^ Hospitals adopted one intervention for one year of the study and then crossed over to the opposite intervention for the second year. A waiver of consent for the initial enrollment was chosen for MINVI due to the lack of a clear recommendation for umbilical cord management of non-vigorous infants and the inherent difficulty in identifying the 3% of term infants predicted to be non-vigorous at birth, prior to delivery. ^8^ Additionally, it would likely have been stressful for expectant parents to be approached for a study where it would be unlikely that their child would be non-vigorous at birth and it would be unethical to add additional time for a patient to be randomized at birth when rapid decision for cord management would be needed.

For the MINVI study, parents were approached after delivery to inform them that their child had received one of the study interventions per protocol. At that time the provider and/or research staff carefully reviewed the study intervention and data collection, and parents gave consent at that time for their child to be included in the ongoing data collection and follow-up. One concern voiced by some IRBs was that parents might react poorly to being approached after the delivery and hearing their newborn was included in a research study without their prior consent. There has been significant debate on whether a waiver of consent is appropriate in neonatal trials.^9^ While IRBs may be reluctant to expose neonates to the potential risks in a trial without parental consent, an argument can equally be made that many more patients are being exposed to the comparable risks of idiosyncratic practice variation as part of routine care. As part of this discussion, it is important to obtain parental perspectives. Therefore, we sought to determine perspectives from the parents whose infants were enrolled in the MINVI study.

## Methods

Six out of ten centers (Sharp Mary Birch, Sharp Grossmont Hospital, University of Utah, University of Alberta, Loma Linda University, and Dalhousie University) obtained IRB approval to send an anonymous survey request to families enrolled in the MINVI trial. No identifying data about the patient or center were collected. We sought to determine whether parents understood the study, had enough time to think about whether to consent to the study, had their questions answered by the research staff, and/or found the waivered approach and the study design to be acceptable. After completion of the study, participants were emailed a copy of the study publication and an anonymized link for participation in the survey once. As no identifiers could be collected (email responses), no incentives were offered. Study data were collected and managed using REDCap electronic data capture tools hosted at Sharp HealthCare. The survey questions are shown in Table 1 and Table 2. No identifiers were collected from participants to ensure they could be anonymized.

## Results

Only consented families at the 6 IRB-approved sites were contacted, excluding those who had a neonatal death (N = 4), leaving the total number of potential respondents to be 1074.

Of the 121 parents who participated in the survey, 116 (94%) parents agreed, 85 (70%) strongly agreed, that they were comfortable with the study design and would participate in a similar study in the future. Additionally, 73 (60%) thought it had a positive impact on their child’s health, and 5 (4%) felt like it had a negative effect. Approximately, one-third (43 parents or 36%) were ambivalent. Of the 121 parents, 115 (92%) found it acceptable to be approached after delivery and the majority (> 90%) of participants thought that information received in the postnatal conversions were sufficient to glean enough information about the study, ask questions, and decide whether to consent or not for further data collection.

## Discussion

Parental perspectives on trials involving waiver of consent are critical to ensure that parental rights and autonomy are maintained. Prior surveys from other countries have shown mixed opinions regarding waiving consent for trials. O’shea et al. conducted a survey in Ireland where 76% of parents were not in favor of a waiver of consent.^10^ Burgess et al. conducted a similar study in Canada comparing prospective consent (before the intervention) or post-hoc consent (after the intervention) with 93% of parents not in favor of the latter approach. ^11^ However, none of these families who completed surveys were involved in a waivered trial.

In a trial of central line catheters in 14 hospitals in Wales and the UK, families of children that were admitted for an emergency provided deferred consent at a higher rate than those admitted on an elective basis (84% vs 69%).^12^ Community consultation for a study of epinephrine use in the pediatric ICU (PICU) setting demonstrated similar parental attitudes with 91% of respondents feeling the use of exception from informed consent was “somewhat” or “completely acceptable” and 74% of respondents indicating that they would be at least somewhat likely to allow their child to participate. ^13^ In a prior survey of families approached with either waived or antenatal consent, conducted in the United States, 69% preferred the waivered approach.^14^ Our survey provides important additional data that suggests that parents are accepting a waived consent process for initial study enrollment and randomization, especially in the setting of an emergent need such as a delivery room intervention.

An important limitation of this study is that we did not have expanded qualitative data from families, in particular, those with more negative survey responses. As part of the initial email, parents were given the results of the trial. Since the trial was not blinded, parents may have been influenced by which arm their child was enrolled into and the trial results. We did not approach those families whose child had died or families who refused to participate after being informed of the study procedures. While these families represent a small proportion of the MINVI cohort (N = 11/1201), they may have provided an important and opposing perspective. We did approach families who had children with poor outcomes such as developing hypoxic ischemic encephalopathy or long-term impairment. Finally, responding families may have different maternal and neonatal characteristics compared to the general population. Weiss et al. demonstrated differences in race/ethnicity, Medicaid status, reported income, perception of illness, and trust in medical researchers in families that refused participation in a newborn trial requiring prospective informed consent.^15^ These issues need to be better addressed during the development of neonatal trials and consents in the future.

The second limitation of this trial is the limited number of responses collected. While to our knowledge this represents one of the largest number of surveys of parents whose child was enrolled in a waiver trial, we had less than a 20 percent response rate. It is unclear whether those families who did not respond would have had a favorable response to our questions.

The results of this study and others described above are encouraging for researchers hoping to conduct clinical trials under a waiver of informed consent; however, a handful of studies does not make a consensus and more work is needed to understand whether acceptance of a waiver of informed consent is generally acceptable or only in certain circumstances. Additionally, the regulations requiring informed consent only allow waivers when obtaining prospective consent would be impracticable (https://www.fda.gov/media/106587), thus even general acceptance of the practice by the community would not give researchers the ability to request such a waiver in all cases. Lastly, researchers who commonly work in fields where such waivers are necessary (e.g. emergency medicine, obstetrics, trauma, etc.) may be well served by engaging in prospective community consultation in conjunction with their IRBs or similar bodies. Such prospective work on the part of the research enterprise could help local IRBs be more comfortable with issuing such waivers when the regulatory criteria appear to be met and ethical questions about the waiver remain.

Although it is not always possible to obtain consent from parents in a waivered consent setting, parental input in the design and conduct of these trials is crucial. Our survey results are limited to this type of interventional study where the inclusion criteria are unpredictable, and the intervention must occur within seconds. Parents were critical members in the design of MINVI.^7^ At the lead center for MINVI (Sharp HealthCare) a parent sat on the ethics review board, and two others sat on the steering committee and the DSMB (Data Safety Monitoring Board) ensuring parental input at all stages of the trial. Per the guidance of our parent members, information about the trial was posted in OB offices and in labor and delivery rooms to give parents the opportunity to opt-out as well. More research into how to support parent decision-making in the setting of waivered trials needs to be explored as well.

## Conclusion

Most parents in our study agree that this type of study was acceptable, understandable and if approached again they would participate in a similarly designed study. While it is reassuring that many parents had a positive response, our survey suggests that a small percentage of parents still have concerns. Continued collaboration with parent groups and institutional review boards are needed to properly develop waivered trials in neonatology.

## Figures and Tables

**Table 1:** Parent Perspective on Informed Consent Experience

	Not Sure	Strongly Agree	Agree	Disagree	Strongly Disagree
I understood the information that I received from the doctor/research staff about the Umbilical Cord Study	3 (2)	67 (55)	46 (38)	3 (2)	2 (2)
I had enough time to think about whether to consent for my child’s data to be used in the study	3 (2)	66 (55)	49 (40)	1 (1)	2 (2)
My questions regarding the study were answered by the research staff or doctor	5 (4)	67 (55)	42 (35)	5 (4)	2 (2)
I found it acceptable to be approached after delivery for participation in the study	4 (3)	70 (58)	41 (34)	3 (2)	3 (2)
If I had another child, I would feel comfortable participating in a study of a similar design	4 (5)	85 (70)	31 (24)	0	1 (1)

All respondents to the survey were birth mothers or fathers.

A total of 121 respondents included from Sharp Mary Birch Hospital, Sharp Grossmont Hospital, University of Alberta Hospital, University of Utah Hospital, and Dalhousie University.

**Table 2:** Parent Perspective on Future Study Participation

	Strongly Positive	Positive	Neither Positive nor Negative	Negative	Strongly Negative
What impact do you feel participation in this study had on your child’s health?	35 (29)	38 (31)	43 (36)	5 (4)	0 (0)

All respondents to the survey were birth mothers or fathers.

A total of 121 respondents included from Sharp Mary Birch Hospital, Sharp Grossmont Hospital, University of Alberta Hospital, University of Utah Hospital, and Dalhousie University.
